# A qualitative study of home health care experiences among Chinese homebound adults

**DOI:** 10.1186/s12877-021-02258-y

**Published:** 2021-05-13

**Authors:** Rui Zhou, Joyce Cheng, Shuangshuang Wang, Nengliang Yao

**Affiliations:** 1 Centre for Health Management and Policy Research, School of Public Health, Cheeloo College of Medicine, Shandong University, 44 Wenhuaxi Rd, Jinan, 250012 Shandong China; 2 NHC Key Lab of Health Economics and Policy Research (Shandong University), Jinan, 250012 China; 3 University of Virginia, College of Arts and Sciences, Charlottesville, VA USA; 4 Department of Gerontology, University of Massachusetts Boston, Boston, MA USA; 5 School of Medicine, University of Virginia, Charlottesville, VA USA; 6 Home Centered Care Institute, Schaumburg, Chicago, IL USA

**Keywords:** Homebound, Home health care, Healthcare experience, Qualitative, China

## Abstract

**Background:**

Home health care services (HHC) are emerging in China to meet increased healthcare needs among the homebound population, but there is a lack of research examining the efficiency and effectiveness of this new care model. This study aimed to investigate care recipients’ experiences with HHC and areas for improvement in China.

**Methods:**

This research was a qualitative study based on semi-structured interviews. Qualitative data were collected from homebound adults living in Jinan, Zhangqiu, and Shanghai, China. A sample of 17 homebound participants aged 45 or older (mean age = 76) who have received home-based health care were recruited. Conceptual content analysis and Colaizzi’s method was used to generate qualitative codes and identify themes.

**Results:**

The evaluations of participants’ experiences with HHC yielded both positive and negative aspects. Positive experiences included: 1) the healthcare delivery method was convenient for homebound older adults; 2) health problems could be detected in a timely manner because clinicians visited regularly; 3) home care providers had better bedside manners and technical skills than did hospital-based providers; 4) medical insurance typically covered the cost of home care services. Areas that could potentially be improved included: 1) the scope of HHC services was too limited to meet all the needs of homebound older adults; 2) the visit time was too short; 3) healthcare providers’ technical skills varied greatly.

**Conclusions:**

Findings from this study suggested that the HHC model benefited Chinese older adults—primarily homebound adults—in terms of convenience and affordability. There are opportunities to expand the scope of home health care services and improve the quality of care. Policymakers should consider providing more resources and incentives to enhance HHC in China. Educational programs may be created to train more HHC providers and improve their technical skills.

**Supplementary Information:**

The online version contains supplementary material available at 10.1186/s12877-021-02258-y.

## Background

The term homebound status typically refers to individuals who are unable to leave their homes or require substantial support to do so, due to their physical or medical limitations [1]. Homeboundness is most prevalent among older adults and the morbidity rate increases with age [2]. The homebound population is increasing in China, as the proportion of older adults is rapidly growing [1, 3, 4]. According to a 2013 survey of urban older adults conducted in two Chinese provinces, the morbidity rate of being homebound and semi-homebound was 15.5% [2]. The likelihoods of hospital admissions were 50% higher and outpatient visits were 20% higher among homebound older adults than among non-homebound older adults [5]. Homebound adults have many unmet medical needs; meanwhile, they face significant challenges in accessing hospital-based care [6]. High healthcare costs and transportation difficulties were the most frequently reported barriers to healthcare access for homebound older adults [5, 7, 8].

Merely improving the traditional hospital-centered care model will not sufficiently meet the unique healthcare needs of homebound adults. The home health care (HHC) model, which has been adopted by various developed countries, provides comprehensive and continuous physician, nurse, and therapist-led care services at home [9]. Some HHC models involve high-quality and cost-effective clinical services, and have been shown to improve access to healthcare, especially among homebound older adults [10]. Care recipients and family caregivers also expressed increased levels of satisfaction toward the technical and interpersonal skills of HHC providers [11–14].

In China, new forms of home health care services have recently emerged, largely due to government-led initiatives. In 2016, the former National Health and Family Planning Commission approved a policy about the legality of home-based medical care. Subsequently, some cities were issued a notice to encourage medical institutions to carry out home health care services, mainly in the form of “family beds” and “visiting services” [15]. The term “family bed” simply refers to home-based health care and alludes to the fact that many homebound patients may be bedbound. Home health care was the most important priority for community development according to the 2018 People’s Livelihood Survey [16]. Toward the end of 2020, a notice promoting the “strengthening of home health care for older adults” [17] was issued by the National Health Commission, and it clarified the elements and references items of home health care service. Simultaneously, the government strengthened the management of home health care and standardized the behavior of home health care services. To receive HHC, patients must either be approached by their physicians, or they may seek to apply for HHC services. In either case, applicants must be diagnosed and evaluated to determine if they meet eligibility criteria before home health care services can be carried out [17]. Despite these government efforts, the HHC market in China is currently still in its infancy; HHC is limited to a few medical institutions and provides a narrow breadth of medical services [18]. At present, home health care services are carried out in Shanghai Municipality and Shandong Province as well as some other places in China. In this study, home health care primarily referred to skilled care, including nursing care and rehabilitation care, though in some cases, participants did receive support from physicians.

Research focusing on the efficiency and effectiveness of HHC in China is scarce. In this present study, we interviewed homebound participants from three different areas in China who had been receiving HHC, aiming to understand their experiences and attitudes toward HHC, and their opinions regarding positive and negative aspects of current HHC models. Through our qualitative research, we intended to summarize best practices and identify possible areas for future improvement to support the development of HHC services.

## Methods

### Study design and setting

This was a qualitative research study based on semi-structured interviews. Qualitative data were collected from homebound adults (mean age = 76) living in Jinan, Zhangqiu, and Shanghai, China.

### Aim

This study aimed to investigate care recipients’ experiences with home health care and areas for improvement in China.

### Participants

We recruited 17 Chinese homebound adults based on the following eligibility criteria: 1) have received HHC, 2) aged 45 years or older, 3) no obvious cognitive impairment, and 4) able to independently answer questions. We defined homebound status as never or rarely having left home in the last month [19]. Individuals who left home a maximum of once per week could be considered for participation in this study [19]. Exclusion criteria included cognitive disorders such as Alzheimer’s disease and dementia, and unwillingness to participate or to complete the interview. There were only a few institutions delivering HHC; thus, we had to recruit participants through purposive sampling from health care institutions that provided home healthcare or rehabilitation care services. These institutions included two community health centers (in Jinan City, Shandong Province and Dinghai District, Shanghai Municipality, respectively) and one hospital (in Zhangqiu District, Shandong Province). Home healthcare providers were contacted first—they then asked their care recipients if they were willing to participate in the interview. Once participants expressed interest, we contacted them by phone to schedule an interview appointment. In addition, a snowball sampling method was used for recruitment, which involved asking our participants to introduce us to acquaintances who fit the inclusion criteria. We then contacted those older adults to ask if they were willing to participate in this study and made an appointment with them if they were interested. The Ethics Committee of School of Health Care Management of Shandong University supported this research (ECSHCMSDU20190101).

### Data collection

Two primary authors conducted semi-structured interviews with participants in July 2019. One author served as the main interviewer who conversed with participants and the other author was responsible for recording and time management.

Interviews were conducted at participants’ homes. Prior to the interview, we introduced the research content and purpose, as well as confidentiality protections, to participants. They signed an informed consent form if they agreed to participate and granted us permission for recording. The average interview duration was 40 min (range 25 to 63 min). The content of interviews included participants’ demographic characteristics (i.e., gender, age, highest education level, and living status) and health conditions, as well as their perspectives pertaining to HHC. In terms of health conditions, we asked about participants’ chronic diseases, oral medicines, and the impact of being homebound on themselves and their family caregivers’ lives. Regarding HHC, we collected detailed information from participants about reasons why they chose HHC services, the major content of services they received, evaluations of current HHC services, and suggestions for future service development. The interviews were guided by open-ended questions and the interviewer probed for more details when necessary. All the questions were included in the questionnaire (Additional file 1). A total of 17 interviews were completed within 16 days. Compensation of 100 RMB was sent to each participant who completed the interview.

### Analyses

Two researchers agreed to end the participant recruitment process when no novel information seemed to emerge from participant interviews. Interview records were stored on a secured device to fully protect the privacy of participants. Recorded interviews were transcribed verbatim into text by one researcher and double-checked by another researcher to ensure accuracy. To protect participants’ confidentiality, participants were given pseudonyms and transcripts were deidentified. The collected data were analyzed by two researchers to reduce subjective errors, using conceptual content analysis [20] and Colaizzi’s method [21–23]. The seven steps were as follows [21, 24]: 1) Each transcript was read several times by two researchers to become familiar with and make sense of the content in its entirety. 2) Two researchers reread each participant’s transcript critically. The phrases and sentences that directly related to the research objectives were highlighted by both the researchers. The two researchers then compared their work and came to a consensus. 3) Formulated meanings from significant statements were coded into different categories. 4) Categories were grouped into clusters of themes. Thematic clusters that related to a particular issue constituted an emerging theme. Two emergent themes and six thematic clusters were identified in this study. 5) An exhaustive description for each of the two themes was developed. The two themes established the fundamental structure of this phenomenological study. The findings were reviewed by a third, senior researcher with expertise in this field of study, to confirm whether the descriptions accurately reflected the experiences and attitudes of Chinese homebound adults toward HHC. 6) After review by the third researcher, relevant descriptions were further modified for clarity. 7) The researchers shared the fundamental structure statement with participants to confirm whether it accurately captured their experiences.

## Results

### Demographic characteristics

Participants’ demographic characteristics are provided in Table 1. Of the 17 participants, about half (*n* = 9) were female, and a majority was older than 60 years, with an average age of 76 years (range 45 to 94). More than half of the participants had an education level of junior high school or below. Most participants lived in urban communities and with their families—only one participant reported living alone.

**Table 1:** Descriptive statistics of the study sample

Characteristic	Number of Participants	Percent
	(*N* = 17)	
**Sex**		
Male	8	47.1
Female	9	52.9
**Age (in years)**		
40–49	1	5.9
50–59	1	5.9
60–69	3	17.6
70–79	3	17.6
80–89	6	35.3
90–99	3	17.6
**Highest Education Level**		
Primary school or below	4	23.5
Junior high school	6	35.3
High school or technical school	5	29.4
Undergraduate or above	2	11.8
**Area of Residence**		
Urban communities	14	82.4
Rural areas	3	17.6
**Living Status**		
Alone	1	5.9
With others	16	94.1
**Living with comorbidity**	17	100.0

Two emergent themes “health conditions and impacts of homeboundness” and “home health care experiences” were identified in the study.

### Health conditions and impacts of homeboundness

All participants were considered homebound, but their level of homeboundness varied. Among them, 9 were completely confined at home due to severe health conditions and 3 were too scared to leave home because of the risk of falls. The remaining 5 participants could only go out with the assistance of their family members. All participants reported having two or more chronic diseases and taking oral medication. Cardiovascular diseases including hypertension, heart disease, cerebral thrombosis, and cerebral infarction were reported by more than half of the participants. Among the cardiovascular diseases, hypertension was mentioned by 11 participants. In addition, 5 participants reported having metabolic diseases (e.g., diabetes). Participants also reported on disability status. Seven participants were homebound due to physical disabilities, such as partial paralysis, amputations, pressure sores, and broken legs. Every participant took two or more different oral medicines; one 70-year-old participant took as many as ten medications per day.

Participants described how being homebound affected their lives; selected quotations are listed in Table 2. The lives of participants became monotonous and boring due to the limitations of being confined at home. They had to give up previous work or housework as a result of their health conditions. In addition, they had to rely on informal caregivers, often family members, to look after them (quotes 4–6), requiring these caregivers to spend more time and energy on them than before the participants became homebound.

**Table 2:** Homebound Chinese older adults’ quotes on health conditions and the impacts of being homebound

Health condition section	Quotes
Chronic disease and oral medicine status	**1:** “I’m in poor health, I’m old and my whole body hurts. I have to take a lot of medications every day.”
	**2:** “I take this huge container of medications every day. Medications alone cost me over thirty yuan a day. I can’t afford it!”
	**3:** “As one gets older, he becomes like a ‘pill jar.’ I rely on medication to keep myself alive.”
Impact on themselves and their family caregivers	**4:** “I can’t do anything except stay at home. I can’t do any chores. I have to rely on my family to take care of me.”
	**5:** “My health is pretty bad, I have to rely on my husband for care. My husband quit his job to take care of me. Our children live far away from us, they have jobs and children to take care of, it’s not convenient for them to come over here.”
	**6:** “Before I had the amputation, I used to farm or help people manage a tree farm, now I can’t do anything. I’m in poor health now, and my wife has to take care of me.

### Home health care experience

A number of questions were asked to help the researchers understand homebound adults’ experiences with HHC; sample responses are provided in Table 3. Firstly, participants were asked why they chose to use HHC services. The first major reason was that HHC was more convenient than office-based care. The participants had varied degrees of difficulty in leaving home. Twelve participants lived on upper floors of apartments without an elevator. They reported that seeing a doctor in their office was challenging (quote 7). Medical care provided at home greatly benefited homebound populations. The second reason was that receiving care at home reduced medical expenses incurred by the participants. Thirteen participants mentioned that HHC was introduced to them by doctors in the hospital. Depending on the participant’s health condition, HHC could offer chronic disease management or wound care to reduce unnecessary hospital admissions (quote 8).

**Table 3:** Homebound Chinese older adults’ quotes on home health care experiences

HHC services	Quotes
Reasons they accept HHC	**7:** “My apartment is on the fourth floor, the building doesn’t have an elevator. My legs are not good, so I can’t go downstairs at all. It’s too inconvenient for me to get out and see a doctor, so I applied for this (home health care).”
	**8:** “Last time, when I went to the hospital, [the doctor] told me that he could come to my apartment to check my wounds and change my dressings. Since then I don’t go out anymore when it’s not necessary. I’ll go to the hospital only when [the doctor] tells me to go.”
Participants’ positive experiences	**9:** “Of course this (home health care) is very convenient. The stairs in my building are very steep. My son used to carry me down every time. This service (home health care) is quite good, in many ways!” **10:** “I’m so thankful to my doctor. She saved my life. There was a time when she did a checkup for me and she found that my heart was about to stop beating, I didn’t feel it myself, but she insisted on sending me to the hospital. After a pacemaker was put in my body, my heart rate came back to normal. I am grateful for the rest of my life!” **11:** “[The doctor] is exceptionally nice, he treats us (his patients) as if we were his relatives. [The doctor] is a good doctor and every one of us says he is very nice and we all respect him.”
	**12:** “The doctor knows my condition very well and takes great care of me. He always comes to see me and we are like friends!”
	**13:** “Dr. Xu’s acupuncture and electrotherapy were very helpful to my health, they were pretty effective for me. I couldn’t lift my neck or cervical spine before, but after she gave me acupuncture, I was able to lift it a little bit, which is quite good.”
	**14:** “My spouse and I are both covered by the Urban Employee Medical Insurance. We first pay a 300 yuan premium, and then the costs of home visits and medical services are paid by the health insurance account. The insurance reimbursement rate is quite high, so we don’t need to spend that much money.”
Participants’ negative experiences	**15:** “The medical and examination equipment they (the doctors) are using is outdated. The quantity of such equipment should be increased. The more advanced the equipment they use, the more they could help us.”
	**16:** “The community hospital is too crowded, there is not enough space, can my doctor give me an IV at home?”
	**17:** “The doctor’s visits are too short in duration, he just takes a look at me and then leaves, it doesn’t even take ten minutes. I wish the doctor would examine me more thoroughly!”
	**18:** “There are many patients like us but there are only a few doctors providing this service (HHC), older people like us need this service, and we hope the number of doctors who do home visits would increase.” **19:** “The first home-visit provider did not do well. His massage was very painful and made me upset. My heart was very tired and the massage was poorly done. I don’t think his technical skill was good enough or maybe he was inexperienced.”

The scope of HHC services varied in different locations. The services in Shanghai usually included monitoring blood pressure, blood sugar, and heart rate; checking care recipients’ medications; and adjusting their medication dosages based on their health conditions. In Shanghai, the initial patient evaluation and diagnosis were usually carried out by the physicians after accepting and meeting the HHC applicant. Subsequently, nurses became the main providers for those older adults. In addition to providing some of the services available in Shanghai, HHC programs in Jinan also provided traditional Chinese medicine services, such as acupuncture and electrotherapy. Medical care providers and Chinese medicine therapists took turns visiting care recipients’ homes. In addition to nurses, various therapists played an important role in Jinan’s home health care model. In Zhangqiu, HHC was mainly provided by doctors and nurses from the Burn and Wound Repair Center who specialized in wound care and burn management. Services included wound care and dressing changes for patients with various types of pressure sores and trauma.

Furthermore, we asked participants about their attitudes toward the HHC they had received. Both positive and negative aspects were described. Positive experiences consisted of the following aspects: 1) This delivery method was convenient for homebound older adults. Compared to non-homebound adults, homebound adults faced greater challenges in seeing a doctor. For example, they faced physical functional limitations and difficulties caused by their built environment, such as apartment buildings without elevators (quote 9). They tended to take a longer time to reach a hospital. HHC helped homebound older adults access medical care as well as medications. Doctors told the participants how to take and flexibly adjust oral medicine dosages according to their health conditions. Family caregivers could retrieve medicines from the hospital, using a medication list prescribed by their doctor. 2) Health problems could be detected in a timelier manner, because initial diagnoses would be made by doctors at older adults’ homes, Nurses would follow up by visiting participants’ homes diligently—around two times per month or more frequently—and arriving on time to their scheduled appointments. Consistent HHC helped participants maintain a stable health status (quote 10). 3) Home care providers exhibited better bedside manners and technical skills than did hospital-based providers. The excellent bedside manners of providers were highly praised by almost every participant. The providers served participants seriously and responsibly (quotes 11–12). Moreover, rehabilitation therapists had advanced technical skills in administering acupuncture and electrotherapy, which were greatly welcomed by the participants (quote 13). 4) China’s medical insurance, which mainly refers to Urban Employee Basic Medical Insurance (UEBMI), usually covered the majority of the cost of approved HHC services. Beneficiaries pre-deposited a certain amount of money into their health insurance account. Then, the medical expenses of approved HHC services could be deducted directly from the health insurance account and the care recipients would be reimbursed for a majority of costs by their insurance (quote 14).

Participants also mentioned some negative experiences with current HHC services. The most prominent problem was that the scope of existing HHC services was too limited to meet the needs of homebound older adults. The content of services mainly included nursing and rehabilitation or narrow specialist treatment. Certain equipment, such as large devices, could not be carried by providers to care recipients’ homes; thus, more comprehensive physical examinations and procedures could not be performed (quote 15). Some participants asked whether they could receive infusion therapy at home, a service that is not currently possible (quote 16). Another complaint was that the healthcare providers’ visit time was too short (quote 17). Compared to the number of homebound older adults in need, there were too few HHC providers (quote 18). Lastly, participants indicated that healthcare providers’ technical skills varied greatly. Most providers that participants encountered in Jinan did not have specialized training in HHC, and a few recipients felt worse after their treatments (quote 19).

Participants suggested increasing publicity surrounding HHC. Homebound participants hoped that the government would pay more attention to the HHC model. If HHC became more prominent, more providers may feel compelled to engage in this method of healthcare delivery. Participants also hoped more HHC providers would proactively seek out homebound older adults in need of these services. Secondly, participants hoped that the treatment of care providers would be improved and their salaries would be increased. These changes might attract more providers who are willing to serve homebound older adults at home. Thirdly, participants hoped that the scope of services might expand in the future, and that providers would receive better education and training. Participants also wanted HHC providers to use better equipment that might detect acute health problems more effectively. Lastly, the holistic, continuous nature of HHC was mentioned. Participants hoped that during HHC visits, providers could teach care recipients’ family members how to care for homebound older adults at home—for example, how to help homebound adults clean themselves or how to prepare nutritious meals suitable for older adults. More comprehensive care would enhance care recipients’ recovery process.

## Discussion

We conducted semi-structured interviews with homebound adults who had received home health care (HHC), to collect information regarding their experiences and attitudes toward HHC. Homebound participants reported positive opinions of HHC based on its convenience. Due to providers’ regular home visits, participants’ health problems could be detected in a timely manner. The bedside manners and technical skills of care providers were greatly praised by participants. In addition, the majority of the cost of approved HHC services could be covered by medical insurance. However, the limited scope of services, the lack of HHC providers, short visit durations, and inconsistent quality of care were also mentioned by participants.

Our qualitative findings in China are consistent with published literature on the efficacy and efficiency of HHC in other health systems. Most research suggested that HHC was a convenient method of accessing medical services for home-limited individuals [25]. Some literature also showed that care recipients’ satisfaction seemed to be strongly tied to the care services provided and clinicians’ positive bedside manners [13, 14]. Providers who were advocates for the development of HHC were serious about their commitment to serving care recipients and they treated recipients warmly. In addition, participants did not have to worry about the financial stress of approved home health care, because medical insurance covered most of service fees [14]. However, there existed a shortage of HHC providers in China [18, 26]. Our findings from this present study supported these conclusions.

In this observational study, the content of HHC was mainly limited to nursing and rehabilitative care. Based on our participants’ responses, we found that nurses and various therapists were the main service providers in China’s current home health care model. Although occasional physician visits occurred, they did not play a central and regular role in care of this study’s participants. In addition to the forms of care we encountered, which most closely resembled the skilled home health care that can be found in other countries such as the United States, modern HHC services in China also include general medicine, palliative care, hospice, and acute care [18]. The current services do not completely satisfy care respondents’ needs; it is necessary to expand the scope of HHC services in China [27].

Based on the experiences of our study’s participants, HHC visit durations were very short and this may be attributed to there being fewer providers for an increasing number of homebound older adults, or the lack of a uniform standard of service. There is an urgent need for more training for HHC providers. Training local medical providers in home care techniques may be a potential method for developing HHC in rural areas [18, 28]. Other research has found that HHC providers’ technical skills were appreciated by care respondents [14]. Though we found similar opinions in some cases, dissatisfaction towards some providers’ poor technique still existed, as providers’ technical skills in this study varied greatly. Sometimes, participants thought HHC providers were helpful, not because their technical skills were advanced, but because they visited regularly to monitor participants’ health status and detect issues; participants seemed to value their patient-provider relationship. There is still room for improvement in the technical training of HHC providers, especially among those who are not currently skilled physicians and nurses, such as rehabilitation therapists and community health workers [18, 29].

It is important to consider not only the qualifications of HHC providers, but also their motivation and job satisfaction, as those who understand the impact of home care, such as clinicians who were involved in the initial innovation of HHC in China, are likely to feel stronger dedication to their profession and provide better patient care. On the other hand, HHC providers who enter the field later might experience lower job satisfaction and higher turnover rates [30]. They may feel less invested in the HHC model if they did not play a role in its inception; also, the work of community health nurses and other clinicians in China can often be stressful, time-consuming, and challenging [30, 31]. The expansion of HHC must involve educating clinicians about the benefits of HHC and the positive impact they will have on their patients, as well as providing job stability, adequate compensation, and support [31], so they feel compelled to contribute to the government’s vision of further developing and improving HHC nationwide.

This study discussed the best practices, shortcomings, and areas for improvement of HHC in China, from participants’ perspectives. Based on our interviews, the following are some suggestions for our policymakers: 1) Increase the supply of home health care programs and workers. Recruiting retired nurses [32] may reduce the pressures felt by current HHC nursing staff, because nurses played a major role in providing HHC. Additionally, nursing homes, nursing centers, rehabilitation hospitals, rehabilitation medical centers, and hospice care centers should be encouraged to extend their healthcare services from institutional medical services to including home health care services, as suggested by the National Health Commission of the People’s Republic of China [17]. To make full use of home-based care, primary care and health institutions could combine current skilled home health care services with primary care and other specialized forms of care, to provide efficient, personalized, multi-level HHC for older adults. 2) Strengthen the training of care providers. Regular training could be organized to improve professional knowledge and skills according to providers’ and patients’ needs. Providers’ technical skills varied considerably in China because they did not receive specialized training in home care before beginning medical practice. It should be necessary to intern for 2 to 3 months with a HHC practice with experienced providers [18]. 3) Standardize home health care service behavior. During the process of providing care, HHC providers must strictly abide by relevant laws and regulations, department rules, professional ethics, service norms and guidelines, and technical operation standards in order to standardize service behaviors [17]. Only physicians, nurses, and rehabilitation professionals who have at least 3 years of clinical work experience are qualified to provide home health care services [17]. 4) Expand the scope of home health care services. Current home health care services mainly include diagnosis and treatment services, medical care, nursing care, rehabilitation treatment, pharmacy services, hospice care, and traditional Chinese medicine treatments that can be delivered at home [17]. Further multi-level HHC and care collaboration should be explored, such as personalized services that include mental health care or long-term care, depending on older adults’ needs [27]. HHC should deliver more comprehensive services to improve care recipients’ well-being and longevity. 5) Increase the use of information technology. Using telemedicine technology could make HHC more accessible [33]. Innovative home health care service models may optimize workflow by using cloud computing, big data, smart healthcare, mobile internet, and other technology [17].

### Strengths and limitations

This study contributed to the development of China’s modern home health care for the following reasons. This was the first study to explore and compare modern HHC in China’s different provinces and to investigate best practices, shortcomings, and areas for improvement. In addition, this was the first study centering care recipients’ voices and feelings about HHC. We collected suggestions regarding the improvement of HHC directly from participants. Despite this study’s important contributions, two limitations should be highlighted. Our sampling method may have resulted in selection and response bias. Participants were recruited via purposive sampling through community health service centers. In addition to recommending individuals who fit the study’s inclusion criteria, doctors tended to refer people with obvious positive health effects resulting from HHC. Those participants were more likely to have a better relationship with doctors or community health service centers. We made interview appointments with the participants in advance, in order to avoid having healthcare providers present which could cause response bias—specifically, social desirability bias. However, in a few cases, providers were present for a portion of the interview, and participants could have felt more inclined to give a positive evaluation of HHC providers and services. This may happen because participants might worry that negative responses could affect their future care, and because they want their providers to view them favorably. Thus, their answers may not be completely objective or fair. However, in the few cases in which providers were present, providers assured participants that their responses would not affect their subsequent care.

Another limitation was that participants’ evaluations of their experiences with HHC were not unanimous. The content of HHC service in three areas of China were not identical. The technical skills of providers in different areas varied greatly, as did participant satisfaction regarding providers’ skill level. Additionally, some participants received more assistance from family caregivers than did others, which may affect their overall perception of their homebound status as well as their perspectives on HHC. Future studies could investigate HHC services in other areas of China, as well as explore the influence of family caregivers on homebound older adults’ healthcare.

## Conclusion

The home health care model benefited Chinese older adults, particularly homebound adults, in terms of patient-provider relationship, convenience, and affordability. There are still many opportunities to improve the quality of care and expand the scope of home care services; these future improvements in service are eagerly awaited by Chinese homebound adults. Policymakers may consider providing more resources and incentives to enhance HHC practice and delivery in China. Educational programs should be created to train more HHC providers and to improve their technical skills.

## Supplementary Information

**Additional file 1.**

## Data Availability

The datasets in the current study are not publicly available to preserve participants’ privacy. However, a de-identified dataset may be made available upon reasonable request of the corresponding author.

## Abbreviation

HHC: Home health care
