# Supplementary material for: A qualitative study of home health care experiences among Chinese homebound adults
Source: BMC Geriatr. 2021 May 13;21:309. doi: 10.1186/s12877-021-02258-y (PMC8117649; doi:10.1186/s12877-021-02258-y)
Supplement: Supplementary file 1 — Additional file 1. [file 12877_2021_2258_MOESM1_ESM.docx]

**Questionnaire**

**Part 1: Demographic Information**

*Note: Older adults with dementia are not eligible for the interview.*

1. Sex? Male; Female
2. Highest Education Level? Middle school or below; high school/secondary school/technical school; community college; four-year college; graduate school or above
3. In general, how is your health? What health conditions do you have? How many medications are you taking every day? What are the dosages?
4. Living status? Living alone; living with others

**Part 2: Research Questions**

**A. Living Situation**

1. We’d like to understand your ability to leave home; when was the last time you left your home? Where did you go? How long were you gone? How did you get there? Who did you go with? Did you feel any pain when you went outside? Did you enjoy going outside?
2. How does your illness or condition impact your ability to leave home?
3. How has life been after becoming homebound? What do you feel like is your role in life? Do you feel that your illness has changed your lifestyle?
4. What do you feel is the most important thing in your life right now? Are you able to engage in activities you used to enjoy?
5. How did your family treat you after you became homebound? Has your relationship with your family changed? Does your homebound status affect your family’s ability to engage in their normal activities/work?
6. Are there other members of your family or the community who check in on you, or to whom you can turn to when you aren’t feeling well? Under what kinds of situations will you contact the physician? What role does your physician play in your life?

**B. Home Health Care Services that Patient is Currently Receiving**

1. For what reason did you start receiving home health care? Are you able to get the care you need whenever you need it? (Provincial 3^rd^ Hospital programs are by appointment)
2. Can you describe the process of receiving home health care?
3. Which part of the care provided by home care providers do you feel is the best? (For example, care provider’s attitude, their skills, or quality of care, etc.)
4. What home care service helped you the most? What was your favorite home care service?
5. What part of the care you have received do you think needs to be improved and why?
6. Did you feel disappointed about any part of the care you received? What service do you think is the least helpful?
7. Do you think the home care services you have received could be improved? If yes, how?
8. Aside from the services you are currently receiving, are there any other home health services that you are interested in receiving?

**C. Patients’ Health Conditions after Receiving Home Health Care Services**

1. Has receiving home health care affected your mobility and physical activity? Has it affected your symptoms?
2. Did you notice any changes in your emotions or feelings after receiving home health care?
3. Are you able to accomplish things that are important to you now, either by yourself or with assistance?

**D. Financial Burden**

1. How do you currently pay for your home health care? Is the cost of your care a financial burden to you?

**E. Home Health Care Providers**

1. Who are your home health care providers? (For example, doctors, nurses, rehabilitative therapists, etc.)
2. When you are visited by various care providers, what kinds of care do they provide? What is your interaction with them? Can you tell me about your good and bad experiences? Do your care providers support you and your caregivers by providing the information you need to manage your health conditions and wellbeing?
3. Do your care providers support you in improving your physical living environment (for example, rearranging furniture) so you can stay safe?
4. In your opinion, what are the qualities of a good home health care provider?
5. Do you feel like your current home health care provider has enough knowledge and skills needed to provide care for you in your home?

**F. Evaluation of Home Health Care Services**

1. In general, how satisfied are you with the care services you are currently receiving?
2. What are your opinions about the healthcare system in your province? Can you describe the positive and negative aspects of the healthcare system? (For example, basic medical insurance, hospitals, physicians, nurses, medical services, medications, etc.)
3. Is there anything else you would like to tell me?
